# Prognostic factors for the successful conservative management of nonocclusive mesenteric ischemia

**DOI:** 10.1186/s13017-022-00436-w

**Published:** 2022-06-03

**Authors:** Yoko Toda, Shunichiro Komatsu, Yasuyuki Fukami, Takuya Saito, Tatsuki Matsumura, Takaaki Osawa, Shintaro Kurahashi, Tairin Uchino, Shoko Kato, Kohei Yasui, Takaaki Hanazawa, Kenitiro Kaneko, Tsuyoshi Sano

**Affiliations:** Division of Gastroenterological Surgery, Department of Surgery, Aichi Medical University, 1-1, Yazakokarimata, Nagakute, Aichi 480-1195 Japan

**Keywords:** Bowel ischemia, Comorbidity, Microcirculation, SOFA

## Abstract

**Background:**

The criteria for deciding upon non-operative management for nonocclusive mesenteric ischemia (NOMI) are poorly defined. The aim of this study is to determine the prognostic factors for survival in conservative treatment of NOMI.

**Methods:**

Patients with bowel ischemia were identified by searching for “ICD-10 code K550” in the Diagnosis Procedure Combination database between June 2015 and May 2020. A total of 457 patients were extracted and their medical records, including the clinical factors, imaging findings and outcomes, were analyzed retrospectively. Diagnosis of NOMI was confirmed by the presence of specific findings in contrast-enhanced multidetector-row CT. Twenty-six patients with conservative therapy for NOMI, including four cases of explorative laparotomy or laparoscopy, were enrolled.

**Results:**

Among the 26 cases without surgical intervention, eight patients (31%) survived to discharge. The level of albumin was significantly higher, and the levels of lactate dehydrogenase, total bilirubin, C-reactive protein, and lactate were significantly lower in the survivors than the non-survivors. Sepsis-related Organ Failure Assessment (SOFA) score was significantly lower in the survivors than the non-survivors. The most reliable predictor of survival for NOMI was SOFA score (cutoff value ≤ 3 points), which had the highest AUC value (0.899) with odds ratio of 0.075 (CI: 0.0096–0.58).

**Conclusions:**

The SOFA score and several biological markers are promising predictors to determine a treatment plan for NOMI and to avoid unnecessary laparotomy.

**Supplementary Information:**

The online version contains supplementary material available at 10.1186/s13017-022-00436-w.

## Introduction

Nonocclusive mesenteric ischemia (NOMI), an acute mesenteric circulatory disorder without organic obstruction of the main trunk of the mesenteric artery or vein, is associated with an extremely high mortality rate of up to 80% [1, 2]. The pathogenesis of NOMI is characterized primarily by microcirculatory dysfunction of the intestine upon a background of systemic circulatory disorders [3]. Several previous studies identified risk factors for the development of NOMI, including a low cardiac output state, septic or hemorrhagic shock, the use of vasoconstrictive drugs, hemodialysis, dehydration, major thoracic or abdominal surgery, and any critical illness [1, 3–5].

While emergency laparotomy for suspected MOMI has been recommended, the clinical outcomes have not improved as we expected [6–9]. Indeed, it is difficult to determine an appropriate bowel resection line by intraoperative findings due to the potentially multifocal and progressive nature of the ischemic disease. In addition, the surgical stress itself induces the releases of cytokines and catecholamines, potentially exacerbating splanchnic ischemia [10, 11].

Meanwhile, there is a spectrum of severity, from mild mucosal ischemia to transmural bowel necrosis and perforation, in the manifestation of NOMI [3]. Some previous studies showed successful outcomes of conservative treatment for the initial stages, with limited bowel wall ischemia [8, 12]. Nonetheless, the decision-making of conservative treatment for NOMI is challenging. Surgical exploration is often needed for the definitive diagnosis of ischemia and determination of its severity. Furthermore, a delayed decision regarding surgical treatment may compromise the chance for survival.

There have been several studies to evaluate risk factors for the development of NOMI and factors influencing the surgical outcomes [6–9]. However, the criteria for the decision of non-operative management remain poorly defined. Hence, the aim of this study was to determine the prognostic factors for a successful outcome in conservative treatment of NOMI.

## Methods

### Patients

**Fig. 1:**
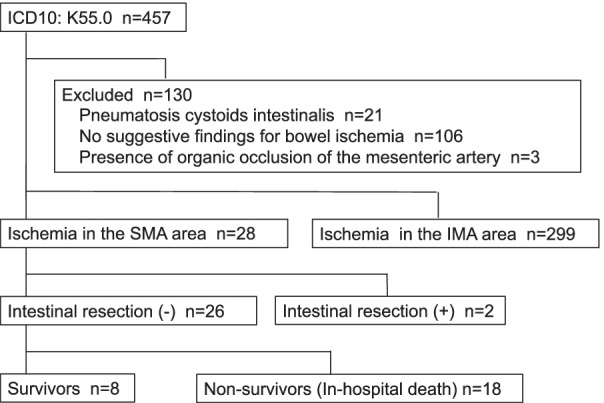
Flowchart of the patient selection in this study. SMA: Superior mesenteric artery, IMA: Inferior mesenteric artery

We identified patients with bowel ischemia by searching for “ICD-10 code K550” in the Diagnosis Procedure Combination database of Aichi Medical University Hospital, between June 2015 and May 2020. A total of 457 patients were extracted and their medical records, including the clinical factors, imaging findings and outcomes, were analyzed retrospectively. Among these, 130 cases were at first excluded because of no suggestive findings for bowel ischemia radiologically or endoscopically (*n* = 106), diagnosis of “pneumatosis cystoides intestinalis” characterized by the presence of multiple gas-filled cysts within the wall of the gastrointestinal tract and pneumoperitoneum without peritoneal irritation [13, 14] (*n* = 21), and the presence of organic occlusion of the mesenteric artery (*n* = 3). Then, 299 cases of ischemic disease, which occurred in the area of the inferior mesenteric artery and were diagnosed as “ischemic colitis”, were excluded. In the remaining 28 cases, diagnosis of NOMI occurred in the area of the superior mesenteric artery was confirmed by the presence of findings for bowel ischemia in contrast-enhanced multidetector-row CT (MDCT), such as attenuated bowel wall enhancement, pneumatosis intestinalis, portal venous gas, ascites, and pneumoperitoneum. In these, two patients who underwent surgical resection of the intestine were excluded. Finally, 26 patients of conservative therapy for NOMI, including four cases of explorative laparotomy or laparoscopy, were enrolled in this study (Fig. 1).

NOMI patients were classified into two groups: group S (*n* = 8), consisting of patients who survived to discharge, and group D (*n* = 18), consisting of patients who did not survive. A variety of clinical factors, including the Charlson comorbidity index [15] and Sepsis-related Organ Failure Assessment (SOFA) score [16], and radiological findings, were compared between the two groups and analyzed for prognosis. The study protocol was approved by the institutional review board of Aichi Medical University Hospital, and informed consent was obtained from all patients.

### Computed tomography protocol

All CT examinations were performed using a 16-detector row CT scanner (SOMATOM Definition AS, SIEMENS, Tokyo, Japan). The technical parameters of CT exams were as follows: tube voltage 100 or 120 kV, tube current determined by Automatic Exposure Control systems, matrix 512 × 512, slice thickness 0.5 mm, collimation 64 × 0.5 mm, pitch 0.8, gantry rotation time 0.5 s, and field of view 30–40 cm. For contrast-enhanced CT, a nonionic contrast agent (350 mg or 300 mg I/mL Omnipaque, Daiichi-Sankyo Pharmaceutical, Tokyo, Japan) was infused rapidly at a rate of 600 mg I/kg for 30 s, using an automated injector.

### Statistics

The statistical analysis was performed using the Statistical Package for the Social Sciences (SPSS) version 25.0 (SPSS, Chicago, IL, USA) for Windows. The Chi-square test or the Fisher’s exact test was used for comparison of categorical variables. Continuous variables were compared using a *t* test when normally distributed, or, otherwise, using the Mann–Whitney *U* test. In all tests, *p* < 0.05 was regarded as significant. Receiver operator characteristic (ROC) curves and the corresponding area under the curve (AUC) were used to evaluate the predictive ability of various factors for the survival in the conservative therapy of NOMI.

## Results

Overall, among the 26 patients with NOMI who did not undergo surgical intervention, eight (31%) survived to discharge. The median duration of hospital stay after diagnosis was 12 days (range: 5–32 days) in the eight patients of the group S. A non-operative treatment with close follow-up was affirmatively indicated for seven of these patients, while one had been considered ineligible for surgical treatment because of severe comorbidity. One patient in group S was administered a vasodilator (Prostaglandin E1). The median survival time after diagnosis in the 18 patients in group D was 2 days (range: 0–40 days). All of the patients in group D had been considered ineligible for surgical treatment, because of severe comorbidities or poor general condition.

There were no significant differences in the patient characteristics (age, gender, presence of comorbidities and Charlson comorbidity index) between group S and group D (Table 1). No significant differences were found between the two groups in the distributions of various comorbidities and acute events before the onset of NOMI, which are presumably potential backgrounds or triggers of NOMI. (The profiles of each individual patient are shown in the Additional file 1.) Table 2 shows the results of blood tests at the diagnosis of NOMI. The serum level of albumin was significantly higher in group S than in group D. The serum levels of lactate dehydrogenase (LDH), total bilirubin (T-Bil), C-reactive protein (CRP), and lactate were significantly lower in group S than in group D. No significant differences were found in the white blood cell count or serum levels of creatinine kinase (CK) and creatinine.

**Table 1:** Patient characteristics

	Group S	Group D	*p*
	(*n* = 8)	(*n* = 18)	
Age, (years)*	81 (71–94)	78 (41–90)	0.504
Gender (male/female)	6/2	13/5	1
*Comorbidities*			
Severe diabetes mellitus, *n* (%)	2 (25%)	2 (11.1%)	0.563
Hemodialysis, *n* (%)	1 (12.5%)	3 (16.7%)	1
Ischemic heart disease, *n* (%)	3 (37.5%)	3 (16.7%)	0.33
Arteriosclerosis obliterans, *n* (%)	0 (0%)	1 (5.6%)	1
*Charlson comorbidity index*			
Comorbidities*	2.5 (1–6)	3 (0–7)	
Age*	4.5 (4–5)	4 (1–5)	
Total*	7 (5–10)	7 (1–10)	0.713

Group S: patients who survived to discharge

Group D: patients who did not survive

*Values are median (range)

**Table 2:** Blood examinations

	Group S	Group D	*p*
	(*n* = 8)	(*n* = 18)	
WBC (×10^3^/µL)	9.0 (7.2–38.3)	9.1 (1.2–18.6)	0.677
Alb (g/dL)	3.5 (2.7–4.3)	2.4 (1.4–6.1)	0.006
LDH (IU/L)	191 (140–262)	384 (146–7400)	0.034
CK (IU/L)	49 (22–176)	195 (12–18,896)	0.097
T-Bil (mg/dL)	0.51 (0.34–0.74)	1.06 (0.4–16.67)	0.005
Creatinine (IU/L)	1.54 (0.84–7.86)	2.20 (0.83–7.7)	0.429
CRP (mg/dL)	0.28 (0.06–9.91)	12.5 (0.24–35.54)	0.003
Lactate (mg/dL)	19.1 (9.1–71.1)	46.0 (14.1–132.9)	0.041

Values are median (range)

Group S: patients who survived to discharge

Group D: patients who did not survive

WBC, White blood cell count; Alb, albumin; LDH, lactate dehydrogenase; CK, creatinine kinase; T-Bil, total bilirubin; CRP, C-reactive protein

Table 3 shows the findings of contrast-enhanced MDCT when NOMI was diagnosed. Attenuation of bowel wall enhancement was observed in all cases. No significant differences were found in the numbers of each CT finding (pneumoperitoneum, ascites, portal venous gas, and intestinal pneumatosis) between the two groups. Six patients (46%) with portal venous gas and four patients (33%) with intestinal pneumatosis survived, whereas no patients with pneumoperitoneum survived.

**Table 3:** CT findings

	Group S	Group D	*p*
	(*n* = 8)	(*n* = 18)	
Attenuated bowel wall enhancement	8	18	1
Pneumoperitoneum	0	3	0.529
Ascites	2	11	0.202
Portal venous gas	6	7	0.202
Intestinal pneumatosis	4	8	1

Group S: patients who survived to discharge

Group D: patients who did not survive

No survivors were found among the patient with critically illness at the onset of NOMI, who had undergone catecholamine administration (*n* = 5), acute blood purification (*n* = 5), and other treatments (*n* = 6, specifically, for brain hemorrhage, recovery after cardiac surgery, severe pneumonia, recovery after surgery for peritonitis, hemorrhage of gastric ulcer and hypothermia with hypoglycemia) (Table 4). Although the number of these treatments for critical illness did not differ significantly between the two groups (Table 4), the SOFA score was significantly lower in the group S compared to group D (Table 5).

**Table 4:** Treatments for critical illness at the onset of NOMI

	Group S	Group D	*p*
	(*n* = 8)	(*n* = 18)	
Catecholamine	0	5	0.281
Acute blood purification	0	5	0.281
Other treatments*	0	6	0.132

Group S: patients who survived to discharge

Group D: patients who did not survive

*Treatments for brain hemorrhage, recovery after cardiac surgery, severe pneumonia, recovery after surgery for peritonitis, hemorrhage of gastric ulcer and hypothermia with hypoglycemia

**Table 5:** SOFA score

	Group S	Group D	*p*
	(*n* = 8)	(*n* = 18)	
PaO_2_/FiO_2_	0	0 (0–4)	
Platelets	0 (0–3)	1 (0–3)	
T-Bil	0 (0–1)	1 (0–4)	
Circulation	0	0 (0–4)	
GCS	0	2.5 (0–4)	
Creatinine	0.5 (0–3)	2 (0–4)	
SOFA score	1 (0–3)	7.5 (1–18)	0.001

Values are median (range)

Group S: patients who survived to discharge

Group D: patients who did not survive

SOFA, Sepsis-related Organ Failure Assessment; T-Bil, total bilirubin; GCS, Glasgow Coma Scale

Table 6 shows the optimal cutoff values of possible predictive factors, estimated by ROC curves and the corresponding AUC. The cutoff value of albumin was 3.3 g/dL, with a sensitivity of 88%, specificity of 83%, and accuracy of 85%. The cutoff value of LDH was 270 IU/L, with a sensitivity of 100%, specificity of 65%, and accuracy of 75%. The cutoff value of T-Bil was 0.74 mg/dL, with a sensitivity of 100%, specificity of 71%, and accuracy of 80%. The cutoff value of CRP was 5.7 mg/dL, with a sensitivity of 88%, specificity of 77%, and accuracy of 80%. The cutoff value of lactate was 24 mg/dL, with a sensitivity of 75%, specificity of 72%, and accuracy of 73%. The cutoff value of SOFA score was 3 points, with a sensitivity of 88%, specificity of 83%, and accuracy of 85%. Table 7 shows the results of univariate analysis of the possible predictive factors for survival. Blood tests (the serum levels of albumin, LDH, T-Bil and CRP) and SOFA score at the onset of NOMI, with the optimal cutoff values, were significantly associated with survival after conservative treatment. Overall, the most reliable predictor of survival for NOMI was SOFA score (cutoff value ≤ 3 points), which had the highest AUC value (0.899) with odds ratio of 0.075 (CI: 0.0096–0.58).

**Table 6:** Optimal cutoff values of possible predictive factors

	Cutoff value	AUC	Sensitivity (%)	Specificity (%)	PPV (%)	NPV (%)	Accuracy (%)
Alb	≥ 3.3 (g/dL)	0.844	88	83	70	94	85
LDH	≤ 270 (IU/L)	0.782	100	65	54	100	75
T-Bil	≤ 0.74 (mg/dL)	0.846	100	71	62	100	80
CRP	≤ 5.7 (mg/dL)	0.882	88	77	64	93	80
Lactate	≤ 24 (mg/dL)	0.77	75	72	55	87	73
SOFA	≤ 3 (points)	0.899	88	83	70	94	85

Alb, Albumin; LDH, lactate dehydrogenase; T-Bil, total bilirubin; CRP, C reactive protein; SOFA, Sepsis-related Organ Failure Assessment

**Table 7:** Univariate analysis of possible predictive factors for survival

Variables	OR	95% CI	*p*
Alb (≥ 3.3 g/dL)	0.035	0.00060–0.41	0.04
LDH (≤ 270 IU/L)	0.091	0.0088–0.94	0.04
T-Bil (≤ 0.74 mg/dL)	0.060	0.0057–0.62	0.02
CRP (≤ 5.7 mg/dL)	0.10	0.015–0.72	0.02
Lactate (≤ 24 mg/dL)	0.21	0.033–1.38	0.11
SOFA (≤ 3 points)	0.075	0.0096–0.58	0.01

Alb, Albumin; LDH, lactate dehydrogenase; T-Bil, total bilirubin; CRP, C-reactive protein; SOFA, Sepsis-related Organ Failure Assessment

## Discussion

Cases of NOMI are increasingly common with the progressive aging of societies during the last decades [17]. Despite a greater awareness of the life-threatening disorder, the diagnosis and treatment of NOMI remain challenging. Explorative laparotomies for the assessment of bowel ischemia, without the performance of any therapeutic procedures, often have been indicated. If the prognostic criteria for the conservative treatment of NOMI patients were to be clarified, the knowledge would be helpful in determining a treatment plan and avoiding unnecessary laparotomy. This study is the first attempt to evaluate the predictive factors which affect the outcomes of non-operative therapy for NOMI. It is noteworthy that 31% of the patients in our study group survived to discharge without surgical intervention, this group included a substantial number of patients ineligible for surgical treatment because of severe comorbidities or poor general condition. Some biological measures of tissue ischemia, cell lysis and inflammation (LDH or CRP), with possibly effective cutoff values calculated, were shown to be promising predictors of survival in conservative therapy for NOMI. Importantly, the index of general condition at the onset of NOMI, represented by the SOFA score (≤ 3 points), was found to be the most reliable factor. The serum levels of albumin and T-Bil were also associated with prognosis and seem mainly to represent the general condition of patients.

The key underlying mechanism for the development of NOMI can be explained by an excessive physiological response to maintain perfusion of vital organs at the expense of mesenteric perfusion, resulting in persistent splanchnic vasoconstriction [3]. According to this hypothesis, the most important treatment for NOMI is to limit the duration and severity of systemic circulatory failure. Fluid resuscitation, with care to avoid overload, is a critical component of the initial care [4]. Administration of vasodilators may be effective for relieving persistent vasoconstriction and improving the prognosis, when the hemodynamics of the systemic circulation are restored, as shown in a previous study [12]. Nonetheless, most of the patients in our series who survived, except for one, did not undergo treatment with a vasodilator.

We should note the heterogeneity in diagnostic methods and criteria for NOMI among previous studies. The diagnostic value of CT has been questioned, and mesenteric angiography or surgical exploration has been considered to be necessary for reliable diagnosis and classification of NOMI [2]. However, recently, the diagnostic precision of CT has been improved with the development of MDCT, in which the resolution of the multi-planar reconstructed images is comparable to that of angiography [18]. In the current study, the diagnosis of NOMI was based on the findings of contrast-enhanced MDCT, such as attenuated bowel wall enhancement, pneumatosis intestinalis and portal venous gas. These findings have been shown to be highly specific to bowel ischemia, despite the sensitivity of detection having been unsatisfactory [17, 19]. Furthermore, the patients with the diagnosis of “pneumatosis cystoides intestinalis” associated with benign prognosis [13, 14], which may include some cases of mild bowel ischemia, were excluded from this study. Therefore, the diagnostic accuracy of NOMI is considered to be reliable in our patient group, although there is the possibility that patients with subclinical or undetected ischemia are missing.

The presence of pneumatosis intestinalis and portal venous gas has been deemed to be definitive findings for severe bowel ischemia, and patients with these signs need urgent explorative laparotomy [9, 20]. Nonetheless, pneumatosis intestinalis is leakage of gas within the bowel wall due to mucosal injury, and portal venous gas is the progression of the pneumatosis intestinalis to the portal venous system [19]. These findings do not necessarily indicate irreversible and transmural necrosis of the bowel. Interestingly, a substantial proportion of patients with these radiological findings survived without surgical intervention in this study.

Our results suggest that NOMI patients with a low SOFA score could be indicated for conservative management. The patients, when supported with non-operative treatment, should be monitored closely by means of serial abdominal examinations, as well as evaluations of their general condition with blood tests. A timely follow-up CT scan may be needed for precise evaluation. Surgical treatment should be considered without delay if the ischemic damage progresses.

The present study had some limitations, it being a retrospective study in a single institution. Although the patients with definitive radiological findings of NOMI according to the uniform diagnostic criteria were selected in our study group, the indication or reason for conservative treatment varied in each individual. In addition, the sample size was too small to perform multivariate analysis. Thus, the possibility cannot be excluded that some confounders may have affected our results. Further studies with larger cohorts are necessary to confirm the clinical relevance of the prognostic factors for the conservative treatment of NOMI.

## Conclusion

In conclusion, the index of general condition, represented by SOFA score and several biological markers (serum levels of albumin, T-Bil, LDH and CRP) at onset, is promising predictors to determine a treatment plan for NOMI and to avoid unnecessary laparotomy. We should note that treatments of NOMI to limit the duration and severity of systemic circulatory dysfunction, such as fluid resuscitation, are important as well as surgical intervention with optimal timing.

## Supplementary Information

**Additional file 1:** Profiles of each individual patient.

## Data Availability

All data generated or analyzed during this study are included in this published article.

## Abbreviations

NOMI: Nonocclusive mesenteric ischemia
SOFA: Sepsis-related Organ Failure Assessment
MDCT: Multidetector-row CT
ROC: Receiver operator characteristic
AUC: Area under the curve
LDH: Lactate dehydrogenase
T-Bil: Total bilirubin
CRP: C-reactive protein
CK: Creatinine kinase
WBC: White blood cell count
Alb: Albumin
GCS: Glasgow Coma Scale
SMA: Superior mesenteric artery
IMA: Inferior mesenteric artery
