# Supplementary material for: Prognostic factors for the successful conservative management of nonocclusive mesenteric ischemia
Source: World J Emerg Surg. 2022 Jun 3;17:32. doi: 10.1186/s13017-022-00436-w (PMC9166604; doi:10.1186/s13017-022-00436-w)
Supplement: Supplementary file 1 — Additional file 1: Profiles of each individual patient. [file 13017_2022_436_MOESM1_ESM.docx]

**Supplementary data**

**Patient profiles**

|  | Age | Gender | Comorbidities | Acute event before the onset of NOMI |
| --- | --- | --- | --- | --- |
| Group S |  |  |  |  |
| 1 | 72 | male | Diabetes mellitus, Ischemic heart disease | - |
| 2 | 94 | male | - | - |
| 3 | 87 | female | Ischemic heart disease | - |
| 4 | 75 | male | Diabetes mellitus, Hemodialysis | - |
| 5 | 83 | female | - | - |
| 6 | 79 | male | - | - |
| 7 | 71 | male | Ischemic heart disease | - |
| 8 | 83 | male | - | - |
| Group D |  |  |  |  |
| 1 | 86 | female | - | Brain hemorrhage |
| 2 | 80 | male | Ischemic heart disease | - |
| 3 | 53 | female | - | Cardiac surgery |
| 4 | 41 | male | - | Severe pneumonia |
| 5 | 85 | male | - | Hypothermia with hypoglycemia |
| 6 | 90 | male | - | - |
| 7 | 65 | female | Diabetes mellitus, Hemodialysis | - |
| 8 | 65 | male | - | Peritonitis |
| 9 | 70 | male | Ischemic heart disease | - |
| 10 | 87 | female | Primary biliary cholangitis | - |
| 11 | 74 | male | Diabetes mellitus, Hemodialysis | - |
| 12 | 70 | male | Hemodialysis, Arteriosclerosis obliterans | Hemorrhage of gastric ulcer |
| 13 | 76 | male | - | - |
| 14 | 83 | male | Ischemic heart disease | - |
| 15 | 86 | male | - | - |
| 16 | 60 | male | Cancer | - |
| 17 | 88 | male | - | - |
| 18 | 88 | female | - | - |

Group S: consisting of patients who survived to discharge

Group D: consisting of patients who did not survive
